# First Africa non-communicable disease research conference 2017: sharing evidence and identifying research priorities

**DOI:** 10.7189/jogh.09.010201

**Published:** 2019-06

**Authors:** Kenneth Juma, Pamela A Juma, Shukri F Mohamed, Jared Owuor, Ann Wanyoike, David Mulabi, George Odinya, Maureen Njeru, Gerald Yonga, on behalf of participants for the first Africa NCD research conference 2017 in Nairobi, Kenya

**Affiliations:** 1 African Population and Health Research Center, Nairobi, Kenya; 2 Clinical Epidemiology Unit, Makerere University, Kampala, Uganda; 3 Aga Khan University, Nairobi, Kenya; 4 African Institute for Health and Development, Nairobi, Kenya; 5 East Africa NCD Alliance, Kampala, Uganda; 6 NCD Alliance Kenya, Nairobi, Kenya; 7 University of Nairobi, Kenya

## Abstract

Non-communicable diseases (NCDs) prevalence is rising fastest in lower income settings, and with more devastating outcomes compared to High Income Countries (HICs). While evidence is consistent on the growing health and economic consequences of NCDs in sub-Saharan Africa (SSA), specific efforts aimed at addressing NCD prevention and control remain less than optimum and country level progress of implementing evidence backed cost-effective NCD prevention approaches such as tobacco taxation and restrictions on marketing of unhealthy food and drinks is slow. Similarly, increasing interest to employ multi-sectoral approaches (MSA) in NCD prevention and policy is impeded by scarce knowledge on the mechanisms of MSA application in NCD prevention, their coordination, and potential successes in SSA. In recognition of the above gaps in NCD programming and interventions in Africa, the East Africa NCD alliance (EANCDA) in partnership with the African Population and Health Research Center (APHRC) organized a three-day NCDs conference in Nairobi. The conference entitled “First Africa Non-Communicable Disease Research Conference 2017: Sharing Evidence and Identifying Research Priorities” drew more than one hundred fifty participants and researchers from several institutions in Kenya, South Africa, Nigeria, Cameroon, Uganda, Tanzania, Rwanda, Burundi, Malawi, Belgium, USA and Canada. The sections that follow provide detailed overview of the conference, its objectives, a summary of the proceedings and recommendations on the African NCD research agenda to address NCD prevention efforts in Africa.

Non-communicable diseases (NCDs) such as cardiovascular disease (CVDs), cancers, chronic respiratory diseases, diabetes and mental illnesses, are a significant and increasing cause of illnesses, disability and deaths globally [1]. For instance, of the 54 · 7 million global deaths in 2016 [2], 39.5 million, or 72 · 3%, were due to NCDs, with majority occurring within low and lower-middle income countries (LLMICs) [1]. Notably, NCDs rise is also faster in lower income countries and populations (the people forming the bottom billion), with an earlier age of onset and higher premature mortalities compared to high-income countries (HICs) [3]. While there has been profound progress made in health of populations over the last three decades, including in the sub-Saharan Africa (SSA) – such as improved life expectancy – the rise of NCDs threatens to reverse these gains and stall social and economic developments [4]. Arguably – NCDs are as much an economic problem, as they are a health epidemic [5]. Cumulative economic losses from NCDs and mental disorders could surpass US$47 trillion (which is more than 75% of global GDP) by 2030 [6]. Recent studies have increasingly shown the intimate link between NCDs and poverty, in which NCDs drain household resources [7], diminish labor supply and productivity [8], as well as impose catastrophic expenditures on poor and uninsured households during treatment of chronic NCD conditions [9]. What is also known is that majority of low income NCD patients tend to use out of pocket payment when seeking medical care [10]. Out-of-pocket payments for health care services drive about 100 million people into poverty every year [11]. Therefore, emerging market economies (such as those in SSA) will be hit harder, even as they continue to grow, if the rising NCD burden remains unchecked [6].

Despite the growing evidence of the health and economic consequences of NCDs in SSA, efforts aimed at prevention and control have been less than optimum [12-14]. Challenges such as acute funding, health system challenges, competing interests from industry actors (such as salt, tobacco and alcohol industries) as well as low NCD awareness levels, remain significant impediments in efforts against NCDs in SSA [15]. Consistent with this evidence, the 2017 WHO NCD Progress Monitor report revealed that less than half of WHO Member States have set NCD targets/indicators to track progress of implementing NCD “best buys” [16]. Also, there are worryingly slow rates of progress at country level in implementing the NCD prevention approaches such as tobacco taxes, strengthening health systems, and restrictions on marketing of unhealthy food and drink products to children [17]. Studies suggest that modest investments to implement cost-effective CVDs interventions in SSA could yield profound reductions in mortalities (from coronary artery disease and stroke) and also reduce economic losses, nevertheless, such opportunities have not been leveraged by SSA governments [18]. Most importantly, since the drivers of NCD epidemic include epidemiological, demographic and economic transitions [19], efforts to deal with NCDs require multisectorial engagement, involving both health and non-health sectors aligning their goals and activities across multiple levels [20].

There are increasing calls to adopt a multi-sectoral approach (MSA) in developing policies and programs to address NCD burden [21]. MSA basically refers to activities undertaken by various non-health sectors to modify health or health-related outcomes or the determinants of health or health equity [22]. However, the mechanisms of MSA application, coordination, and successes in NCD prevention remain poorly characterized in SSA countries [23]. Furthermore, the absence of an active African civil society NCD forum curtails the coordination of NCD prevention research efforts in Africa. While there have been previous attempts to establish an African NCD civil society organizations (CSOs) network, these efforts have been characterized by challenges such as retaining interest of network partners, meeting stakeholder and partner expectations and scarcity of dedicated resources [24,25]. Nevertheless, an African CSOs NCD forum could be a platform to share progress in research, disseminate lessons learnt and replicate them in various other settings, formulation of research priorities and networks of partnerships and collaboration relevant to Africa.

In recognition of the above gaps in NCD programming and interventions within Africa, a three-day NCDs research conference was organized by the East Africa NCD alliance in partnership with the African Population and Health Research Center (APHRC) in Nairobi, Kenya, in January 25-27, 2017.

The conference objectives included:

- to disseminate evidence on the analysis of NCDs prevention policy development and the role of multi-sectoral action (MSA) in NCD policies and programs in five African countries,
- to share progress in NCD prevention and control initiatives (including policies and actions) in select African countries, and
- to identify Africa NCD research priorities and formation of a collaborative research Network.

## PARTICIPANTS

The conference drew more than one hundred fifty participants and researchers from various sectors including the academia, civil society, and state actors – mainly ministers of health, international NGOs, the WHO, health care providers and practitioners of both allopathic and alternative medicine, the private sector and patient support groups. In addition, there were several researchers from several institutes in Kenya, South Africa, Nigeria, Cameroon, Uganda, Tanzania, Rwanda, Burundi, Malawi, Belgium, USA and Canada who attended the conference.

## THE CONFERENCE STRUCTURE AND PROCEEDINGS

Each conference session had a chair and at least two rapporteurs. The sub-theme for the first day was **Multi-sectoral action in NCD prevention and control**. There were key note speeches from the conference hosts namely, representatives from; the EANCDA, the African Population and Health Research Center, and the International Development Research Centre (IDRC) as funders of the conference. Other remarks were delivered by a representative from the World Health Organization-AFRO office and the Kenyan Ministry of Health. Remarks centered on the herculean threat posed by rising NCDs to health of populations and economic development and social well-being, and also highlighted the broad outlook of the NCD response situation in Africa as pertains policy, programs and research.

The first day had a total of eight presentations. Initial presentations reported on the Analysis for Non-communicable Disease Prevention Policies in Africa (ANPPA) project-that sought to analyze the status of NCD prevention policies in five African countries (including South Africa, Malawi, Nigeria, Cameroon and Kenya) and the role of multi-sectoral action (MSA) in the NCD policy formulation and implementation processes. What then followed were highlights on the economics of NCDs control and in particular analysis on restrictions on tobacco and alcoholic beverages in Kenya. The sub-theme for the second day was **NCD prevention and control initiatives**. Introductory remarks were delivered followed by a set of eighteen presentations mainly focusing on the global NCD advocacy and priorities *(perspectives from NCD alliance)*, the WHO-AFRO NCD Monitoring framework, Health metrics in NCD Research, as well as on Poverty and NCDs. Within the same day, presenters shared some of the ongoing NCD research projects across Africa and abroad. Later on, NCD country representatives presented on the status of NCD research, policy and actions in East African countries. On the final day, the conference sub-theme was **NCD research priorities and formulation of collaborative networks**. The first session commenced with a presentation on research regulation in East Africa and funding opportunities available for NCDs research. During the second session, three working groups were formed to address the following questions:

- what should be the priority NCD research themes and agenda in Africa?,
- What are the possible NCD research communication and networking mechanisms in Africa?,
- what is the framework for NCD research collaboration in Africa?

This paper highlights the proceedings of the three-day NCD conference while providing thumbnail summaries of presentations and discussions covering the various aspects of NCDs in developing countries.

### Multi-sectoral Action in NCD Prevention in Africa (ANPPA PROJECT)

There are increasing calls to utilize multi-factorial and multi-sectoral approaches (MSA) in NCD prevention and control efforts [21]. MSA refers to actions undertaken by sectors outside the health sector, with or without collaboration with the health sector, on health or health- related outcomes or the determinants of health or health equity [22]. The high and rising NCD burden and that of its risk factors stems from many social and economic determinants like globalization, trade and marketing, demographic and economic transitions, leading to behavioral and metabolic risk factors [26]. Most of these determinants of health are exterior to the purview of the health sector. This implies that effective NCD prevention and control efforts need to include participation from non-health sector actors (such as trade and commerce, agriculture, education, urban planning, and transportation) [27]. The WHO, through its global strategy for NCD prevention and control, has advocated for a ‘whole-of-government’ approach (in which public service agencies work across portfolio boundaries to achieve shared health goals) and also identifies MSA as critical for NCD prevention at population level. Also, WHO 2008–2013 Action Plan for Prevention and Control of NCDs identified several “best buy” interventions for NCD prevention including measures to reduce the common NCD risk factors. These best buys overlap across several sectors, and their implementation require participation from multiple actors [18,28]. Successful application of MSA approaches has been reported in several high-income settings, for instance – in tobacco control initiatives. However, such evidence on the mechanisms of application and success of MSA for the control of other NCD risk factors (ie, harmful use of alcohol, physical inactivity and unhealthy diets) is very limited in SSA. The “Analysis for Non-communicable Disease Prevention Policies in Africa” (ANPPA) project implemented by APHRC aimed to generate evidence on the extent to which countries have applied MSA in developing policies to prevent NCDs in six SSA countries particularly –Kenya, Malawi, Nigeria, Cameroon, Togo and South Africa. Findings of this study provide a deeper understanding of the processes through which MSA is actualized, including the challenges, constraints and enabling factors [23].

The ANNPA project findings revealed a poor understanding of MSA and how it should be operationalized. Despite the availability of a WHO toolkit on MSA implementation for NCD prevention, few countries have domesticated this document and applied it. In majority of SSA countries studied – questions still linger as to the precise definition and taxonomy of the term “non-state actors” and also the criteria to be applied to identify and ensure broad representation of all sectors in MSA teams. In some countries, there are existing inter-agency committees which form basis for MSA, while for some, MSA participation is based on invitation. Broad involvement of patients, normal citizens, women or consumers in the MSA processes is poor, yet these groups are critical in NCD advocacy beyond government sectors. Case studies from most of ANPPA countries showed that legislation does not necessarily translate into implementation, for example, enforcement of tobacco legislation depends on the security sector, but most often this sector is not included in NCD policy making. Therefore, there is need to shift focus and invest more on effective mechanism of policy enforcement, MSA implementation accountability, and monitoring and evaluation framework. Investment in awareness programmes within sectors is also a critical enabler of MSA.

Conference panelists noted that NCDs need to be reframed not only as a public health agenda but more importantly as an economic, social and development issue. This would most likely achieve greater buy-in from policy makers and other non-health sector actors when communicating NCD prevention needs. In addition, sufficient explanation should be provided to the non-health sectors on the potential benefits of adopting MSA approaches to NCD prevention. Conference panelists pointed out that certain MSA models have been successfully applied to support supply of essential drugs and medicines, there is broad consensus that these could be leveraged for NCD prevention. In terms of MSA implementation – most national ministries of health are not capable of convening inter-agency group meetings, however, the Office of the Presidency has the oversight mandate and capacity and therefore NCD units need to involve the presidency in international treaties targeting NCDs so as to support instrumentation of related treaties and ensure compliance where needed. Also, health sector actors need to participate and be actively involved in tax reforms and budget making processes as it allows for a chance to provide guidance on fiscal measures or influence increased funding for NCD prevention related activities.

Challenges to MSA application identified included sustainable funding which is heavily dependent on donor support, most often directed by donor priorities. Also identified were low levels of knowledge and awareness on MSA among different government departments in various countries. For instance, majority of departments were neither aware of their links to NCDs nor what MSA entails.

Following these findings in select SSA countries, recommendations at country level included; the need to establish an expert working group to address critical issues such as actor mapping process, involvement of patients, women and consumers, and to develop a clear context based definition of MSA. Health sectors should lead in creating awareness for MSA application, nurture and catalyze actions among various stakeholders and define roles for each actor and indicators of monitoring progress in MSA for NCD prevention. Furthermore, depending on each country’s context – there may be prioritization of specific NCDs, build MSA for these priorities before expanding the effort to other NCDs and risk factors. To improve coordination of MSA for NCDs, actors have suggested the formation of a chronic disease commission (a national coordinating body). Having lobby groups in places such as parliaments can also support NCD advocacy efforts. At the regional and international levels, having an international ombudsman to pressure national governments to prioritize NCD prevention and control is preferred. In conclusion, MSA for NCD prevention needs to be dynamic and adjust to the local realities as there is no one size fits all.

### NCD prevention and control initiatives in SSA

#### Estimating the burden of NCDs in SSA-findings from the 2015 Global Burden of Disease study

The burden of NCD-associated morbidity and mortality is heavy and rising rapidly especially in the SSA [1]. From 1990 to 2015, NCD deaths increased from 25% (1.7 million) to 34% (2.7 million) of total deaths in SSA. Majority of these deaths were caused by CVD (12%), cancer (7%), and diabetes (5%) of total deaths [29]. With regards to health loss, CVDs are ranked 6th leading causes of Disability-Adjusted Life Years (DALYs) whereas diabetes, mental and substance use disorders and cancers are ranked 9th, 10th, and 11th respectively. In addition, from 1990 to 2015, the health loss associated with CVDs and diabetes increased by 40% and 46% respectively [30]. Significant differences in prevalence are also witnessed across countries in the SSA. For instance, 7.2 million Nigerians have CVDs compared to 3.7 million Ethiopians and 2.8 million South Africans. About 4 million South Africans suffer from diabetes, this compared to 3.2 million in Nigeria and 2.2 million in DRC [31]. High blood pressure, poor diets, and air pollution top the list of risk factors for health loss from NCDs in SSA [29]. Nearly 6% of DALYs and 15% of deaths in SSA are due to high blood pressure and unhealthy diets. Other metabolic risk factors such as high BMI and high blood glucose are also leading risks of NCDs. Variations in NCD risk factors based on gender are also evident [32,33]. Discussants decried the impact of NCDs in SSA, and the effects it has on low and middle social economic households where the cost of illness not only represents much of the direct costs of medical care, but also has an impact on family disposable incomes. Also noted were the deficiencies in addressing NCDs from basic science research and medical training to health service delivery, population-wide interventions and access to essential medicines and technology in Africa. There was consensus that more efforts are needed to enhance a robust primary health care system that focuses on the social determinants of health, as well as promoting early screening and treatment.

#### World Health Organization Regional Office for Africa (WHO-AFRO) – NCD Monitoring framework

In consolidating NCDs prevention and control as well as improving the coordination of these efforts, several global and regional initiatives have been established. In September, 2011, the United Nations (UN) high level meeting (HLM) of Heads of State and Government met to affirm political commitment against NCDs and prioritized NCDs prevention and control [34]. In 2012, the World Health Assembly (WHA) adopted a global target of 25% reduction in NCDs associated premature mortality by 2025 (25 by 25) [35]. In 2013, the WHA launched the WHO Global NCD Action Plan 2013-2020, that included 9 voluntary global targets and 25 indicators to accelerate national-level efforts to address NCDs [36]. Furthermore, in 2014, a UN General Assembly session was held to assess progress achieved in global NCDs prevention and control efforts and the progress to achieve set targets by member countries was noted as good. In same year (2014), the outcome document of the UN HLM of the General Assembly reiterated the same roadmap of national commitments, including four time-bound commitments such as setting national NCD targets for 2025 or 2030 and monitoring results by 2015; developing a national multi-sectoral action plan by 2015; implementing the “best buy” interventions to reduce NCD risk factors by 2016 and to strengthen health systems to address NCDs. There are also a set of ten progress monitoring indicators designed to track progress in every country [37] (see **Box 1**).

BOX 1 **Ten progress Monitoring Indicators (Adapted from the World Health Organization, 2015) [37]**

1. Set National NCD targets and indicators
2. Accurate mortality data by each country
3. Conduct Risk factor surveys utilizing the standardized STEPwise survey methodology.
4. Develop a National integrated NCD strategy and action plan
5. Set measures to regulate tobacco demand by using taxation, smoke-free policies, health warnings and bans
6. Reduce harmful use of alcohol using available regulations, pricing policies, advertising and promotion bans
7. Reduce exposure to unhealthy diet using salt/sodium regulation policies, saturated fatty acids and trans-fats policies, and marketing to children restrictions, restrict marketing of breast-milk substitutes.
8. Public awareness on diet/physical activity
9. Guidelines for the management of major NCDs
10. Drug therapy/counseling for high-risk persons

The Sustainable Development Goals (SGDs) agenda were unveiled in 2015. Goal 3 aims at health and welfare. NCDs are particularly captured in several strategic objectives such as reduction of NCD related mortality through health promotion, NCD prevention and treatment of common NCD risk factors, reduction in deaths and injuries from road traffic accidents. The SDGs also call for expansion of universal health coverage (UHC), strengthening and implementation of the World Health Organization Framework Convention on Tobacco Control (WHO FCTC), support of research and improvement of access to affordable essential medicines and vaccines, substantial increase in health financing and human resource capacity, and elimination of all forms of violence against women and girls [38]. Conference participants acknowledged the existing frameworks that support the coordination and monitoring of efforts to address NCDs. While NCD prevention and control progress indicators in most African countries remain suboptimal, the WHO AFRO NCD monitoring framework serves to raise awareness and reinforce political commitment.

#### Challenges experienced in prevention and control of NCD

Despite the considerable progress made in developing and adopting a global NCD strategy, several countries in SSA have been slow to translate the global strategy into comprehensive national policies and plans, thereby affecting their effective implementation. Other factors that impede efforts to address the NCD burden include; a) incomplete NCD burden data which is grossly underestimated because NCDs such as sickle cell diseases, mental illness, injury and suicide are poorly documented and not included in the reported data, b) low (even though increasing) knowledge and awareness on NCD burden, drivers and impact hinders attempts to adopt healthy behaviors and also the uptake of early screening and treatment of NCDs such as cervical cancers and hypertension, as well as adherence to treatments to achieve blood pressure control, c) the national policies and plans for the prevention and control of NCDs are often underfunded, d) the non-availability of relatively inexpensive medications used for managing chronic NCDs in public sector facilities is a key challenge limiting the ability to address effectively the management of NCDs, poverty, which is widely common among residents of LMICs, including SSA plays a fundamental role in the onset, progress and mortality of NCD patients. Majority of individuals with chronic diseases in these low resource settings resort to out-of-pocket payment, which then drives the already poor to more abject poverty. While the social and economic implications of NCDs remain scarcely characterized in most SSA countries, this information is important as it may attract prioritization of NCDs by all relevant sectors, and raise the political commitment needed to effect national and regional action against NCDs. Also, there is constant interference from the commercial and economic interests of tobacco, alcohol and food industries making it difficult to control their activities that continue to drive key NCD risk factors. Conference discussions focused on identifying opportunities to address these challenges, and suggestions included that high-level policy makers should prioritize NCDs and provide adequate funding to national policies and plans aimed at promoting healthy lifestyles and that of prevention and control of NCDs. Also noted, was the need for increased research and reporting of socio-economic consequences of NCDs among the poor and vulnerable, and the application of innovative and sustainable solutions that can be incorporated as part of broader development frameworks.

**Poverty and NCDs – Case study from Rwanda.** Conference presentations highlighted trends in urbanization across several towns in Africa, and mostly characterized by increased rural-urban migration [39], and significant changes in the patterns of health and disease [40]. Other factors such as emergence of slums, overpopulation, and urban poverty have aggravated health statuses of individuals in SSA [41,42]. Perhaps the most at risk groups to NCDs in the next decade will be the urban poor, who thrive within complex nutrition, food systems as well as physical activity environment [43]. Most urban settings face issues such as insecurity concerns, lack of organized markets, inadequate resources for physical activity, and poor housing (risking indoor pollution)- all promoting risks for NCDs. Significant proportions of the population in these urban poor settings have limited healthy lifestyle options [44]. As observed in Rwanda in 2015, NCDs were responsible for 35% of all deaths, 52% of all outpatients’ consultations and 22% of all hospitalization in district hospitals. The greatest contributors of NCDs associated DALYS in Rwanda included congenital anomalies, unipolar depression, asthma, epilepsy, chronic obstructive pulmonary disease (COPD), diabetes, cervical cancer, and rheumatic heart disease [45]. While the NCD burden in most HICs is driven by traditional risk factors (ie, obesity, tobacco use, and other factors collectively termed “poor lifestyle” choices), Rwanda’s NCD epidemiology is significantly linked to malnutrition, infections, and toxic environments- factors all exacerbated by poverty [46]. Similarly, individuals exposed to NCD risk factors increase across the decreasing economic strata [47]. In addition, individuals of lower socioeconomic status (SES) tend to have limited access to health services, including screening, early diagnosis and treatment, as well as poor adherence to health promotion messages and therapy. NCDs may drive individuals and households to poverty through loss of earnings and reduced productivity [48], as well as catastrophic health care expenditures [48,49]. Increased recognition of the role of poverty in NCDs has accelerated efforts to re-framing NCDs and injuries for the poorest billions in LLMICs [5]. Nevertheless, in Rwanda, interventions to address poverty and NCDs are vanishingly scarce. The Rwanda Economic Development Poverty Reduction Strategy (EDPRS II) (2013-18) have an overarching goal of poverty alleviation, while the national NCD policy targets creation of health promoting environments, support to community actions to reduce exposure to modifiable NCD risk factors and injuries, as well as strengthen and mainstream NCDs programs within the national health systems. However, there is no cooperation links between these generalized efforts to address poverty on one hand and NCDs on the other. Despite the existing link between poverty and NCD, global funding for health and NCDs is tilted in favor of the rich countries with 60% of total funding going to upper-middle income countries, 35% to lower-middle income countries and only 5% to low-income countries [50,51]. Therefore, the countries with the largest burden of disease receive the least amount of financial support to address NCDs.

#### Examples of ongoing NCD prevention and Control Projects

Researchers shared examples of ongoing initiatives undertaken to prevent and control NCDs in the various SSA countries, including those focusing on: (i) legislation (eg, to control tobacco and alcoholic beverages), (ii) models of NCD care (eg, integration of NCD primary care to strengthen health systems), (iii) advocacy to improve access to essential medicines and technologies for NCDs, and (iv) policies and direct actions to modify NCD risk factors. Key highlights of these ongoing NCD projects are as shown below.

**Research to guide practice: Enhancing HIV/AIDS platforms to address NCDs in low-resource settings (PEPFAR-NCD project).** The remarkable progress made in confronting the global HIV epidemic offers profound opportunities to address the increasing threat of NCDs in people living with HIV (PLHIV) and across general populations. However, questions remain about how to enhance the HIV platforms to deliver integrated HIV and NCD care to PLHIV in SSA. The PEPFAR-NCD project involved researchers, policy implementers and government representatives to explore approaches to improve evidence necessary to enhance the gains in HIV/AIDS prevention and control while addressing rising burden of NCDs. The National Institutes of Health (NIH), with leadership from the Center for Global Health Studies (CGHS) at Fogarty, implemented the project in collaboration with the President's Emergency Plan for AIDS Relief (PEPFAR) and over 70 active participants. While the primary focus of this project was to expand care for chronic NCDs for people living with HIV (PLWH), the ultimate goal is to generate evidence supporting investments to have health care systems that are comprehensive for all prevalent health needs in low-resource settings. So far, the project has delivered outputs including; i) landscape analysis through examining peer-reviewed and grey literature on integration of priority NCDs into existing HIV health systems and care protocols in LMICs, in addition to identifying a possible HIV/NCD research agenda for low resource countries, ii) conducted a workshop in Washington DC, USA of researchers, national program implementers and policy makers from eight countries in SSA, and research organizations in the US, to explore existing models on integrated HIV and NCD care, iii) supported a call for HIV and NCD data modeling proposals to develop approaches to estimate the burden of NCDs (such as cervical cancer, diabetes, depression and cardiovascular disease) in PLHIV in SSA. More recently, the project has supported development of 11 supplement articles to be published in the AIDS journal. These set of eleven supplement papers provide greater details on various integrated HIV/NCD components (such as policy, NCD medicines supply chain, economics of integration and global partnerships).

**Healthy Heart Africa.** Healthy Heart Africa (HHA) program has committed to tackling hypertension and the raising burden of cardiovascular diseases (CVDs) in Africa. Funded by AstraZeneca, the program aims to reach up to 10 million hypertensive patients across Africa by 2025. It spreads across 24 counties in Kenya, and there are plans to expand to other countries in Africa. While the HHA program anticipates the significant barriers to access of CVD prevention and management care, it uses a model with three key pillars including: i) Increasing education and awareness on hypertension, its risk factors and available interventions for prevention and treatment, ii) conducting training of health providers (including nurses) while driving care to lower levels of health care system, and support development of guidelines appropriate for community-based implementation, iii) facilitating access to essential medicines and technologies (EMTs) by developing supply chain and distribution models that improve access to low cost, high quality branded anti-hypertensives. To enable the building of local capacity – the program has joined forces with six local partners across the private, public and faith-based sectors in Kenya at every level of care, to create a demonstration model that works for the country. HHA also uses collaborations with Kenya’s Mission for Essential Drugs and Supplies (MEDS), to establish secure supply chains for anti-hypertensive medicines, and with Savannah, a Kenyan data management company, to allow for continuous monitoring of program outputs and patient level data capture.

**Integration of NCD primary care into HIV – pilot project in Kenya.** NCD primary care services in most low resource settings including Kenya are still nascent, compared to other more established health service delivery platforms (such as HIV/AIDS, Maternal and Child Health (MCH), and Family Planning (FP)). There has been increasing interest to leverage on the existing resources within these well-established disease platforms, to achieve enhanced NCD prevention for populations in Kenya. However evidence is scarce on how exactly to implement these attempts. The NCD care integration pilot project, supported by the Grand Challenges Canada was implemented by the Aga Khan University, Kenya. The project implemented systematic NCD screening (ie, risk factors for CVDs, chronic obstructive pulmonary disease, cervical cancers) among routine patients attending the HIV clinic and other primary health care (PHC) units (such as mother child health (MCH) and family planning (FP) clinics) at a rural sub county hospital in Kenya. Findings from the NCD integration into PHC settings revealed increased knowledge and awareness on NCDs among existing PHC staff and patients, improved skills in NCD care, improved NCD and risk factor detection, improved ability to offer NCD counselling and referral services even within lower cadre facilities. Similarly, a study on NCD/HIV integration by the Medicines Sans frontiers (MSF) in Kibera slums (Nairobi) and in Embu, revealed that integration improved detection of NCD cases within routine HIV clinics, and also increased capacity by health staff to attend to more new cases. With the increase in research and data on feasibilities of HIV and NCD integration including some positive results from the pilot studies in Kenya, Uganda, and South Africa – there are calls to reorient health systems to address the prevalent heath needs of primary care attendees. Such results can be used to inform resource allocation (personnel and funding), and planning in the health sector for NCDs (ie, resources to follow needs). Nevertheless, not all attempts to integrate NCDs produce positive results, indeed integration into other vertical platforms may carry along risks such as dilution of outputs from high profile vertical disease programs, such as HIV programs. Further research is needed to build more evidence on what can be integrated, and how integration can better be done, as well as outcomes of integration on other programs, on NCDs services, on staff effectiveness and to answer cost-effectiveness questions.

**Strategies for access to Essential Medicines and Technologies (EMTs) for NCDs – Advocacy in NCD.** The situation in LMICs is that most essential drugs for NCDs (eg, diabetes) are rarely available in 80 percent of public or private facilities, and are least available in the public sectors, rural areas, and at the lowest levels of care. What is also known, for instance in Kenya, is that prices are higher in private health facilities than public facilities. Moreover, different counties have varied infrastructure levels for access to essential medicines and technologies (EMTs) for NCDs. These and several other factors inevitably restrict overall access to essential medicines and technologies (EMTs) to manage NCDs. Moreover, in most countries, drug stocking is not need-based, and allocations is not informed by client affordability. Several models of interventions have been attempted by organizations such as PATH International, to improve access to EMTs including, raising awareness of availability and affordability of EMTs for diabetes, and inspire a wide range of stakeholders at the global and national levels to act and address this challenge. While recognizing that the current approaches and systems for procurement and distribution of diabetes EMTs are neither efficient in most of SSA, nor are they meeting existing needs, there is need for proper financing which includes dedicated budget for NCDs; strengthening of Health system to include NCD in all primary care settings and formulation of standard guidelines; supply chain management improvements; improvement in policies and governance.

**Tobacco and alcoholic beverages control in Kenya – Economics of NCD control.** Implementing increased taxation and restrictive policies on unhealthy products, such as tobacco, alcoholic beverages and sugar sweetened drinks, can produce major health gains for the poorest in society. Though regulation on sugary drinks has been limitedly implemented, this year, five countries, including Ireland, the United Kingdom and South Africa, will join 26 nations who have raised prices of sugary drinks. Kenya is among the LMICs countries which has implemented increased taxation on tobacco (from around 2005/2006) and regulation of alcoholic beverages by an act of parliament in 2010. An analysis of this tax and price regulation in Kenya revealed that it led to higher levels of alcohol and tobacco abstinence compared to other countries in the region. Taxation on these products implies availability of a huge potential market for these products in Kenya. Most importantly, taxation alone as a health promotion intervention, has limited reach in minimizing exposure to alcoholic substances since majority (more than 58%) of the alcoholics consume home brewed alcohol in Kenya. Nevertheless, taxation remains an important regulatory tool that needs to be extended on sugar sweetened drinks. There are suggestions to standardize tax on alcoholic drinks and base it on the alcoholic content in a given beverage rather than volume of the whole beverage as is the current practice. This would raise taxes on spirits since they have higher alcoholic content. However, caution should be exercised in this regard since increased taxation of alcoholic drinks could trigger a shift by most consumers to home brewed alcohol (which is unregulated), thus rendering the tax policy ineffective. Ironically, one approach to decrease consumption of home brewed alcohol is to reduce the cost of beer. Stability of tax regime is needed to in order to allow assessment of impact of taxation on alcohol and tobacco in a given setting. Also, there is need to explore additional mechanisms to bend downwards the demand curve for tobacco and alcohol use.

### NCD research priorities and collaborative networks for Africa

#### Research regulation – using the East African Context

Regulations in medical research is guided by principles including; respect for persons (autonomy), beneficence and justice which are operationalized in ethical guidelines. Ethical guidelines set standards for the conduct of research and contributes to the quality and consistency in ethical review. While there are modest growth seen in ethical reviews for research in the East African region, there are increasing demands by researchers to improve on the process and time taken to approve research proposals. There is need for cooperation between different committees in the review of multi-center studies (especially those involving more than one country), requiring more than one review for these multiple reviews to occur effectively. Cooperation of different ethical review bodies across different countries shortens time for review and administrative quality control is embedded in products of such cooperation. Available modes of cooperation include having joint committees (integration), regional committees (joint review) and collaboration. Also, the main opportunities for integration of the review process exist in international frameworks such as the “WHO, UNESCO Universal Declaration on Bioethics and Human Rights” and using the regional cooperation frameworks such as the East African Cooperation Protocol and the African Union.

#### Research funding opportunities for NCDs in Africa

Funders present at the conference included, the International Development Research Centre (IDRC), GlaxoSmithKline (GSK), Astra Zeneca, and Novo Nordisk. They all shared their respective funding opportunities relevant to NCDs, while highlighting their priority program areas and ongoing projects being supported. On its part, Astra Zeneca dedicates approximately US$4.9 billion in Research & Development across five countries, with focus on CVDs & metabolic diseases, oncology, respiratory, inflammation & autoimmunity diseases, and supports local academic medical research in NCDs. Some of the projects currently being supported include, *“Healthy Heart Africa (HHA)”* program and the *“Young Health Programme”* which focuses on NCD health promotion among adolescents. In addition, *“phakamisa”* is AstraZeneca's access program to increase capacity of health care professionals to diagnose and treat breast and prostate Cancer. Novo Nordisk focuses in diabetes care and other chronic conditions such as growth disorders and obesity. Together with its partners, Novo Nordisk has aimed to develop scalable and sustainable solutions that enhance access to diabetes care by building health care professionals’ capacities and providing better access to chronic disease care in 22 African countries. The IDRC’s 2015 - 2020 focus is on food, environment and health programmes. With regards to NCDs prevention, IDRC supports food systems research for prevention of food-related chronic diseases and tobacco control research. IDRC has also funded research aimed at understanding the economics of NCDs prevention and control interventions, such as research in South Africa to explore the economic arguments for and against legislative and fiscal policies to improve nutrition, economic modelling of NCD burden and innovative financing mechanisms for health. GlaxoSmithKline (GSK) conducts research, develops and manufactures innovative medicines, vaccines and consumer health care products. It supports the Africa NCD open Laboratory programme, which is a virtual research and development laboratory that shares research expertise and resources with funders, researchers and stakeholders to conduct NCD clinical & translational research in Africa.

#### NCD research themes and priorities in Africa

The causes of NCDs are multifaceted and include social, economic and even cultural drivers, and similarly, opportunities for intervention and research exist at various levels such as individual, community and national/international levels. Interventions can be at various stages such as at health promotion and prevention, treatment, care and rehabilitation. Conference participants developed a broad range of research priorities to form the basis for the regions response to tackling the rising burden of NCD, as detailed in **Table 1**.

**Table 1:** Non-communicable diseases (NCD) research themes and priority projects

NCD themes	What the theme entails	Priority projects
**a) NCD Prevention and treatment**	Efforts to; understand social causes of NCDs, improve access to medication, adherence, and health services, behavior change for NCD prevention, prioritize NCD “must buys”. Improve access to rehabilitation/referral services	1. Situational Analysis – data available, policy – evaluation 2. Implementation, monitoring and evaluation of “must buys”
**b) NCD data and surveillance**	Efforts to build health information systems to collect quality and routinely update data on NCD associated Morbidity and mortality.	1. Assessing the availability, quality, accuracy and completeness of data already collected 2. Improve mechanisms of conducting sentinel or routine risk factor surveillance
**c) Health systems research**	Efforts to understand and improve how health services are organized to achieve health goals, and how different actors interact in the policy and implementation processes to contribute to policy outcomes.	1. Assess models of health system integration2. Assess effectiveness of chronic care models 3. Innovative ways to improve funding, payment and access to medicines, vaccines and technologies to remote populations
**d) Economics in NCDs**	Efforts to quantify the cost of NCD burden, cost of NCD interventions; cost of inaction; cost of various service delivery models, understanding the politics of resource allocation in health; economics of sin taxes, innovative financing, health insurance and minimizing out of pocket payments.	1. What is the cost-effectiveness of the NCD “best-buy” interventions 2. Explore the politics of health resource allocation 3. Effects of NCD interventions on behavior change for NCD prevention
**e) NCD policy research**	Understanding the impact of NCD related policy on prevention of diseases, effects on other sectors, implementation researchers	1. Assessing the impact of NCD policy 2. Mechanisms of MSA applications in policy formulation

#### Framework for collaboration and research coordination in Africa

**Figure 1:**
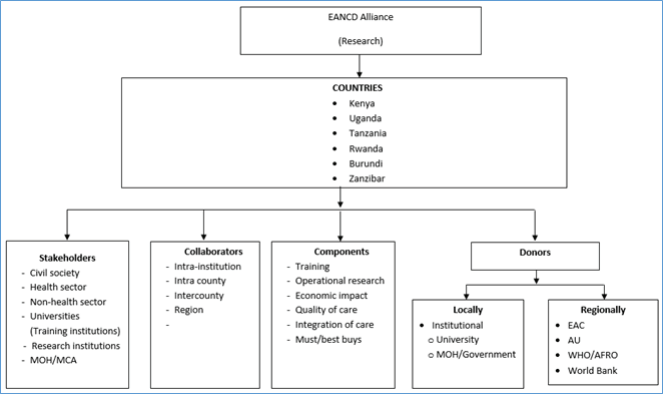
Framework for non-communicable diseases (NCD) research coordination: the case of East African region.

Participants of the conference discussed relevant multidisciplinary networks and coordination frameworks for NCD research and modalities of collaboration between NCD researchers that allows for maximum leveraging of existing resources within African countries and support the NCD control efforts. These initiatives would be channeled through existing regional networks or country alliances (**Figure 1**).

Modes of research collaboration between these institutions and groups (seen in **Figure 1**) included; a) submission of joint proposals, b) engaging in capacity building through exchange programmes, c) conducting multi-country comparative projects, and d) sharing research outputs, expertise for implementation and conferences, and workshops.

***Research communication and network.*** Conference participants proposed structure and mechanisms of communication and sharing of research opportunities and outputs. An organizational framework and a platform for communication and resource sharing was generated as illustrated below.

Organizational Structure: a three-tier structure was modelled along the African NCD research network resolutions developed in Mauritius in 2016. The tiers included a;

- Continental committee of five leading NCDs researchers
- Regional committee made up of representatives from the East, West, Central, North, and south regions of Africa
- National committees based at country levels

Modes of Communication: the modes used for communication and sharing of NCD research progress and outputs within the African region included:

- An African NCD Network Website
- Quarterly newsletters
- Africa non-communicable disease journal
- Social media channels such as Facebook, Twitter, LinkedIn, Youtube channel, mySpace, Flickr views

## CONCLUSIONS

Overall, the first Africa NCD conference was successful in managing to draw consensus among the delegates on how to address rising burden of NCDs through research interventions, set priorities and collaborations. Conference participants agreed on the need to reframe NCDs not only as a public health agenda but more importantly as an economic, social and development issue. This would improve buy-in from other non-health actors and adoption of MSA approaches to NCD interventions. In addition, there is need to define and expand the composition of MSA teams to include a wider representation. There are many existing multidisciplinary networks that simply require a coordination framework for NCD research and modalities of collaboration between NCD researchers in SSA. This coordination and leveraging of strengths would allow for maximizing of resources within and across countries and create synergy in NCD prevention and control efforts.

Furthermore, resources and capacities in the south and north can be shared for improved research outputs. Key modes of research collaboration between these institutions would be: a) submission of joint proposals, b) engaging in capacity building through exchange programmes, c) conducting multi-country comparison projects, and d) sharing research outputs, expertise for implementation and conferences, and workshops.
